# Plug cartilage tympanoplasty in children

**DOI:** 10.1016/S1808-8694(15)30150-6

**Published:** 2015-10-18

**Authors:** José Arruda Mendes Neto, Felipe Costa Neiva, Fábio Brodskyn, Marcel das Neves Palumbo, Ana Cláudia Valério Bittar, Roberta Novaes Borges Petrilli, José Ricardo Gurgel Testa

**Affiliations:** a MD. Otorhinolaryngology resident - UNIFESP-EPM; b MD. Otorhinolaryngologist. Fellow at the Department of ENT - UNIFESP/EPM; c MD. Otorhinolaryngology resident - UNIFESP-EPM; d MD. Otorhinolaryngology resident - UNIFESP-EPM; e MD. Otorhinolaryngologist. MSc. Student - Department of Otorhinolaryngology - Head and Neck Surgery - UNIFESP/EPM; f MD. Otorhinolaryngologist. MSc. Student - Department of Otorhinolaryngology - Head and Neck Surgery - UNIFESP/EPM; g PhD, Adjunct Professor - Department of Otorhinolaryngology - Head and Neck Surgery - UNIFESP/EPM

**Keywords:** cartilage, tympanic membrane, tympanoplasty

## Abstract

The treatment of tympanic membrane perforations in the pediatric population with sequelae of chronic otitis media represents a challenge to otolaryngologists. **Aim**: to assess the clinical and audiometric results of the inlay technique with a tragus cartilage plug. **Materials and Methods**: we assessed 23 patients (ages between 1 and 15 years) who underwent plug tympanoplasty. Study design: clinical retrospective. **Results**: repair success rate was of 82.6%, with audiometric parameters improvement in 87.5% of the patients. Complications were minimum. **Conclusion**: considering the results attained, this method of tympanoplasty should be considered a good treatment option for tympanic membrane perforation in children.

## INTRODUCTION

The first known attempt to repair an ear drum perforation was allegedly made by Banzer, in 1640, using a swine bladder membrane graft1. In 1853, Toynbee introduced the “artificial eardrum”, a small rubber disk with a silver rod - to facilitate introduction and extraction^1^, ^2^, ^3^. In 1878, Berthold, used a skin graft and in 1887, Blake recommended the use of a piece of paper as a template to tympanic membrane regeneration1. Notwithstanding, it was only after 1944, with the beginning of antibiotics and with the improvement in surgical techniques that other materials were used as grafts in tympanoplasties^1^, ^2^. After this time, in 1952, Zollner and Wullstein published their methods, using retroauricular skin grafts; however they did not succeed in treating tympanic membrane perforations^1^, ^2^, ^4^, ^5^, ^6^.

Brazilian data, published by Costa in 1976, showed good results in the treatment of tympanic membrane perforations with the use of temporal fascia grafts and dura mater grafts^7^. In 1983, Miniti et al., showed a significant audiometric improvement in patients submitted to tympanic membrane repair surgery with the use of dura matter^8^. In 2003, Oliveira et al. Observed that the use of synthetic biomaterial (latex biomembrane with polylysine) could contribute to a greater graft take rate of temporal fascia in tympanoplasties^9^.

Thus, numerous types of tympanoplasty grafts have been described. The most commonly used techniques for graft placement on the tympanic membrane are the “underlay” (medial) and “onlay” (lateral), and the most used types of graft are the temporal muscle fascia and perichondrium, with similar success rates (approximately 90%)^4^, ^5^. Among children, these rates vary between 66 and 93.5% with the use of temporal fascia graft^10^. However, these two techniques require a skin incision in the external acoustic meatus (EAM), which causes greater morbidity and the need for better post-op care, reducing the use of these techniques in children^6^, ^11^. In 1989, Gross described the “inlay” approach, with the use of a fat tissue plug for small perforations, however without success in repairing the perforations^12^.

The cartilage was first used to rebuild the ossicular chain in 1958, by Jansen^13^. Some years later, this material started to be used as a graft in tympanoplasties, especially in cases of advanced middle ear diseases, because of their robustness, offering greater resistance to resorptions^4^. In 1998, Eavey described tympanoplasty in children using the tragal cartilage and bilateral perichondrium (cartilage plug) and the placement of a graft without incisions in the EAM (inlay approach) ^11^. This new approach, when compared to the previous ones, showed a number of advantages for its use in children: no need for a tympanic-meatal flap, reducing pain and the need for post-op care; possibility of placing the graft in the so called “hostile” ear drums (those with tympanosclerosis plaques and malleus exposure); shorter surgical time, it can be carried out under general anesthesia without orotracheal intubation, thus giving the patient the possibility of an early hospital discharge - and reducing costs - no need to dress the EAM, since the graft fits stable onto the ear drum^6^, ^11^.

The goal of the present investigation is to assess the clinical and audiometric results of the inlay approach with tragus cartilage plug to correct tympanic membrane perforations in the pediatric population.

## MATERIALS AND METHODS

We carried out a retrospective study, through the analyses of medical charts from 23 patients with tympanic membrane perforations, operated from October of 1999 through September of 2005. All the patients had the following characteristics: 1- age below 16 years, 2- central perforation with possibility to visualize all its borders, 3-tympanic membrane perforation caused by chronic otitis media, 4- disease in one ear only, 5- conductive hearing loss with air-bone gap below 50 dB.

The patients were broken down in two groups: age below or equal to 10 years (n=11), and one group of older patients (n=12), since the rate of upper airway infections is considered higher in the first group.

All the patients were assessed through a clinical history, general and otorhinolaryngological physical exam, audiometry, image exams (temporal bone CT scan) when necessary and preoperative tests. The mean value of tonal audiometries was calculated using the values from 500Hz, 1kHz, 2kHz, 4kHz in a study of the air and bone conduction curves.

The microscope used in the surgical procedure had a video camera coupled to it, allowing for high quality documentation of the procedure. Through this latter resource, it was possible to measure perforation size in percentage through the following calculation: perforation area /total area of the tympanic membrane X 100.

Cartilage plug tympanoplasty postoperative results were evaluated as to perforation closure, need for further surgeries, hearing improvement checked by means of the audiometry and the presence of postoperative complications.

Patients were observed for an average time period of nine months after surgery, varying between three to twelve months.

Statistical analyses were carried out using the chi-square with correction by Fisher”s exact test.

This study was analyzed and approved by the Research Ethics Committee of our institution, under protocol # 20070820101245.

## TECHNIQUE

**Figure 1:**
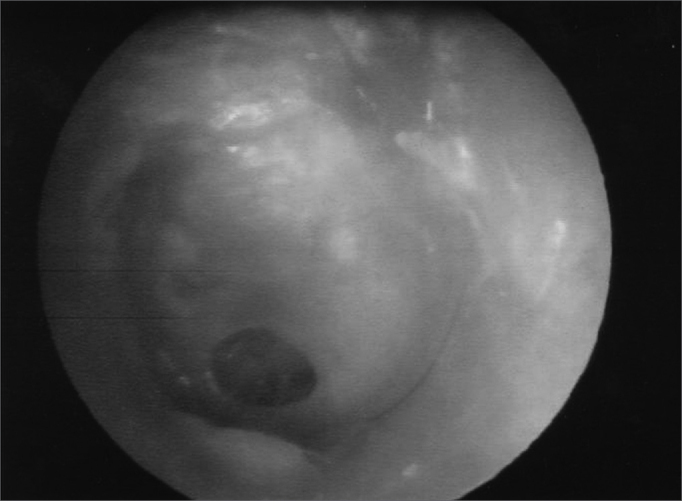
Central perforation in the tympanic membrane.

With the patient under general anesthesia and under microscopic view, we examined the tympanic membrane perforation (Fig.1). The perforation border was removed with a straight tip stylet and, later on, a curved tip stylet was used to scratch the mucosal face of the tympanic membrane. The perforation”s size and shape were measured with the help of a hook-type stylet, drawing its shape in a piece of sterile paper (nylon suture wire wrapping which was used later to close the tragal incision -donor site). The paper was cut and the perforation size was checked against the paper mold created.

A small, one centimeter incision, was made across the tragus (15 scalpel blade). We used a delicate scissors to dissect the tragus inferior cartilage preserving the perichondrium in both sides, and we removed a fragment slightly larger than the mold we had previously created. This incision was sutured up with 5-0 nylon wire.

**Figure 2:**
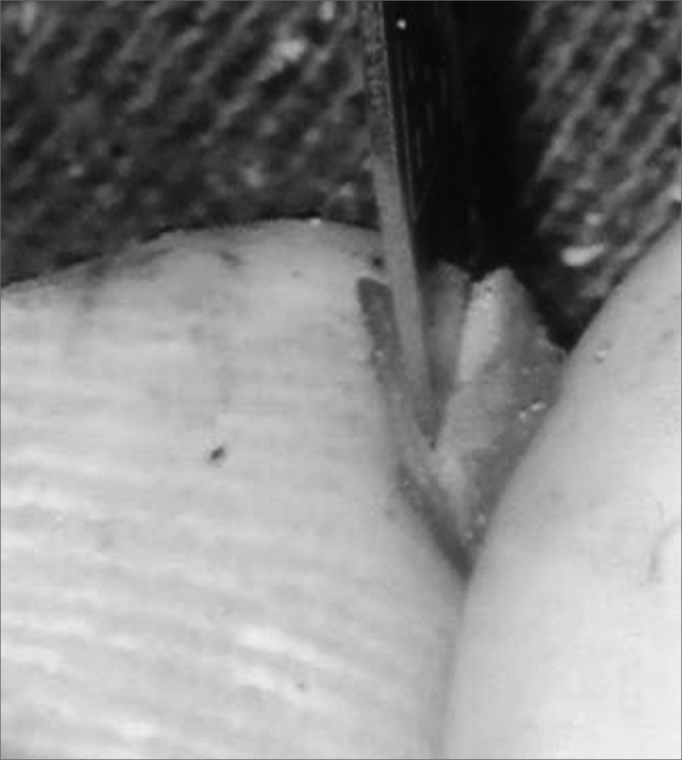
Creating a sulcus in the cartilage graft.

**Figure 3:**
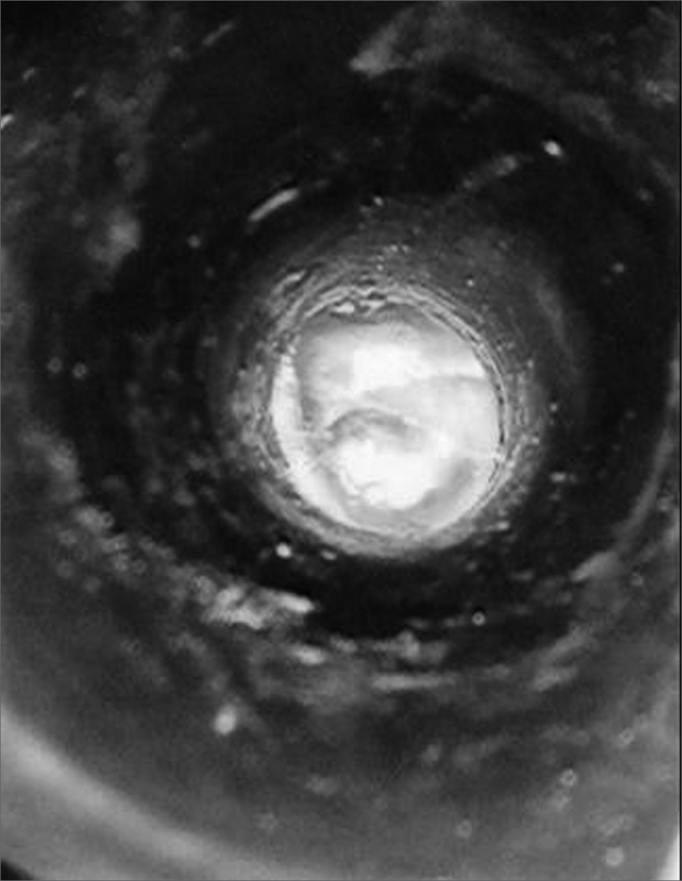
Plug fitting perfectly on the ear drum perforation.

Using the same #15 scalpel blade, we cut the entire perimeter of the graft, making a small 2mm-deep sulcus in it (Fig.2). Following that, the graft was placed on the perforation with perfect fitting. (Fig.3). On top we placed a thin gelfoam layer with antibiotic ointment, and a cotton ball was used to seal the EAM.

**Figure 4:**
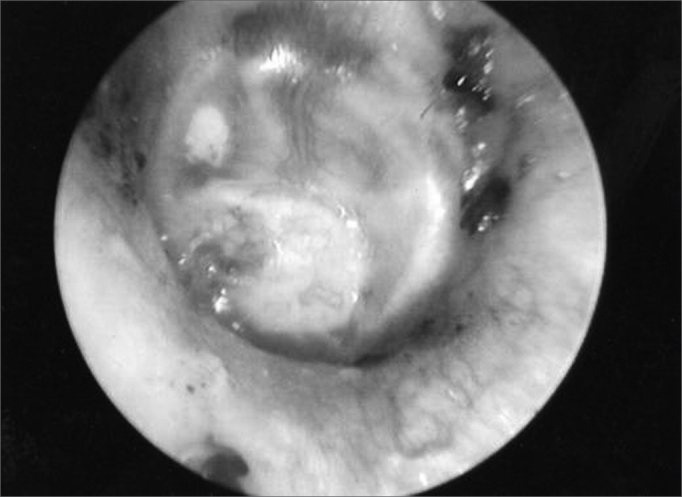
Otoscopic view one week after surgery.

With one week of post-op, the suture was removed and the gelfoam fragments were aspirated, allowing us to see the graft and its viability^2^, ^4^, ^11^(Fig. 4).

## RESULTS

From a total of 23 patients submitted to tympanoplasty with cartilage plug, 14/23 were males (65.4%) and 9/23 (34.6%) were females, with ages ranging from 3 to 15 years, with mean value of 10.3 years. In the group of patients between one and ten years of age, there were 8/12 (66.7%) males and only 4/12 (33.3%) females (Table 1). The left side was involved in 10 cases (43.4%), while the right side was affected in 13 cases (56.6%).

**Table 1:** Patients” traits.

	1-10 years group	11-15 years group	Total
Number of patients	11	12	23
Gender			
males	5	8	14
females	5	4	9
Age (years)			
mean	7,3	12,8	10,3
Variation	(3-9)	(11-15)	(3¨15)
Perforation side			
Right	5	8	13
Left	6	4	10

30 cartilage plug tympanoplasties were carried out in these 23 patients, and in seven of them (30.4%) it was necessary to reoperate in order to close the perforation, and in six of them the reoperation was successful. Of these seven, only one was between 1-10 years of age. Mastoidectomies were also necessary in order to open the antrum and attic in six individuals (26.1%).

Perforation sizes varied from 15% to 90%. In the 1-10 year-olds this variation was of 15 to 25% (mean value of 29.3%), while in the other group this variation was higher (25 to 90%), with a mean value of 48% (Table 2).

**Table 2:** Perforation and air-bone gap in the two groups studied.

	1-10 years Group (n= 11)	11-15 years Group (n= 12)
Perforation size		
Mean	29,3%	48%
Variation	15-25%	25-90%
Preoperative gap		
Mean	16,3dB	26,6dB
Variation	5-30dB	5-50dB
Postoperative gap		
Mean	5dB	12,4dB
Variation	0-15dB	0-30dB
Gap improvement	11 (100%)	9 (75%)

In the initial auditory evaluation, we observed a minimum air-bone gap of 5dB and maximum of 30dB in the group with 1-10 years of age (mean value of 16.3dB) and, in the final postoperative evaluation, a minimum difference of 0db and maximum of 15dB (mean value of 5dB). In the other group, the preoperative values varied between 5dB and 50dB (mean value of 26.6dB). In the postoperative there was a variation of 0 to 30dB (mean value of 12.4dB). In three patients there was no gap improvement with tympanoplasty, all of them belonged to the group of older patients (Table 2).

Among the patients submitted to the procedure, we observed one postoperative complication - this individual had local infection with improvement of signs and symptoms through clinical measures, however the perforation remained, without gap improvement.

There was total perforation closure in 19 patients (82.6%). We observed residual perforation in four patients (17.4%) during the follow up period, and half of them belonged to the group of younger patients. There was no statistically significant difference between the two groups studied (Table 3).

**Table 3:** Residual perforation after tympanoplasty in the two groups studied.

	1-10 years group (n= 11)	11-15 years group (n= 12)
Residual perforation after tympanoplasty	2 (18,2%)	2 (16,7%)

Fisher”s exact test: p-value=0.67

The age range did not impact air-bone thresholds after surgery, as seen on Table 4.

**Table 4:** Age range influence on the air-bone gap improvement after tympanoplasty.

	1-10 years group (n= 11)	11-15 years group (n= 12)
≤15dB gap after tympanoplasty	6 (54,5%)	3 (25%)
≤15dB gap after tympanoplasty	11 (100%)	9 (75%)

Fisher”s exact test: p-value=0.43

And finally, tympanoplasty was effective in improving the gap alone in each group, according to the table below (Table 5).

**Table 5:** Results from tympanoplasty according to age groups.

	1-10 years group (n= 11)		11-15 years group (n= 12)	
	gap ≤15dB	gap >15dB	gap ≤15dB	gap >15dB
Before tympanoplasty	6 (54,5%)	5 (45,5%)	3 (25%)	9 (75%)
After tympanoplasty	11 (100%)	0 (0%)	9 (75%)	3 (25%)

Fisher”s exact test: p-value=0.01 (1-10 years Group)

P-value=0.01 (11-15 years Group)

## DISCUSSION

Cartilage, as well as fascia, vein and periosteum are mesenchymal tissues and, for this very reason, they do not scale off. They have no secretory glands, nor hair follicles as those found in the skin, thus being used as tympanic membrane graft without the risk of causing iatrogenic cholesteatomas ^1^.

Contrary to other materials, cartilage has some physical properties that facilitate its use in tympanoplasties. These grafts are nourished by diffusion and easily incorporated on the tympanic membrane, which has been confirmed in second look tympanoplasties^4^, ^5^, ^6^. It is a more robust material, easier to fit on the ear drum perforation site1. It is thicker, less prone to resorption and retraction^4^. Nonetheless, the cartilage acoustic transfer characteristics are theoretically worse because of its thickness^2^, ^4^, ^6^. In 2000, Zahnert et al. carried out an experimental study concluding that a 500μm-thick cartilage has an acceptable acoustic transfer capacity with good mechanical stability^14^.

Success rates of tympanic membrane perforation closure with cartilage plugs in adults are high. In 2000, Testa et al., published a closure success rate of 96.8% with hearing improvement in all the cases^2^. Lubianca-Neto et al. in 2000, published rates of 90% and 94.4%, respectively^6^. These are the goals to be reached in children.

Notwithstanding, low immunity, high upper airway infection rate and Eustachian tube dysfunction are factors responsible for reducing the success rates of tympanoplasties in the pediatric population^3^.

In the present investigation we found a total perforation closure in 19/23 patients, with a success rate of 82.6%. In the younger group, this rate was of 81.8%, while in the other group it was of 83.3%. These rates are similar to the ones found by Hennawi in 2001 and Couloigner et al. in 2005^3^, ^10^. There was no statistically significant difference between the two groups studied (p>0.05). This fact is probably due to the larger size of perforations in the older group, while in the younger group there are more factors inherent to their age range that impair graft take after surgery.

Twenty patients (87.5%) had hearing improvement after tympanoplasty. In the youngest one there was a mean gap reduction from 13.6dB to 5dB, with threshold improvement in all the patients. In the other ones, there was a mean gap reduction from 26.6dB to 12.4dB, with threshold improvements in 9/12 patients. The other three patients remained with bone-gap differences equal to the ones they had before surgery. These results are similar to the ones published by Couloigner et al. in 2005^10^. Therefore, regardless of the group studied and despite the cartilage graft density, hearing results are very good (p<0.01).

Of the seven patients who were reoperated, there was perforation closure in six of them, and only one case belonged to the 1-10 year-olds group. This shows that such procedure bears good results both in primary surgeries as it does with reoperations.

The smaller perforations, the shortest preoperative gaps and the high perforation closure success rates and hearing improvement indicate that this type of approach can be carried out as early as possible to treat sequelae of chronic otitis media in the pediatric population. Moreover, differently from other approaches, this one does not require major postoperative care and cause less pain because it does not require the physician to dissect a tympanic-meatal flap. This makes the tragus cartilage plug procedure a good option for the treatment of ear drum perforations. Complications were rare in some of the papers published, as they were in our study^2^, ^4^, ^11^.

Perforation closure success rates are similar to those achieved with other materials used, such as the temporal fascia. There was no statistically significant difference as to the closure of perforations and hearing improvement between cartilage plug tympanoplasty and that carried out with temporal fascia in the study published by Couloigner et al. in 2005^10^. Eavey in 1998 and Levinson in 1987 also showed success rates very similar to these when other materials were compared to cartilage plug^4^, ^15^.

## CONCLUSION

1. Tragus cartilage plug tympanoplasty was an effective approach to close tympanic membrane perforations in 82.6% of the patients from a pediatric population.
2. There was hearing improvement in 87.5% of the individuals.
3. Age range did not have a significant influence on the clinical and audiometric results obtained after surgery in the two groups studied.
